# Translational medicine as a permanent glue and force of clinical medicine and public health: perspectives (1) from 2012 Sino-American symposium on clinical and translational medicine

**DOI:** 10.1186/2001-1326-1-21

**Published:** 2012-10-05

**Authors:** Jiebai Zhou, Duojiao Wu, Xinqing Liu, Shuoqi Yuan, Xiaoqiu Yang, Xiangdong Wang

**Affiliations:** 1 Department of Pulmonary Medicine, Fudan University School of Medicine, Zhongshan Hospital, Shanghai, China; 2 Biomedical Research Center, Fudan University School of Medicine, Zhongshan Hospital, Shanghai, China; 3 Center of Academicians, Shanghai, China

## Abstract

**Abstracts:**

Health systems globally face challenges and opportunities in balancing quality, access, and cost, where clinical and translational medicine (CTM) should play more important and powerful roles in the identification, development and validation of solutions and strategies. Strategic collaboration can gather global strengths and resources and improve health systems, care delivery, regulations and policies. CTM-driven innovation and development has the potential to achieve step-change improvements across three dimensions. Thus, we have the reasons to believe that CTM will play even more roles in the development of new diagnostics, therapies, healthcare, and policies and SAS-CTM will become more and more important platform to obtain the latest development in CTM internationally and explore new opportunities in the international collaborations.

## Introduction

Clinical and translational medicine (CTM) has been highly emphasized since Dr. Elias Zerhouni, the former director of the National Institutes of Health, proposed “The NIH Roadmap” in 2003 [1]. CTM is an emerging area comprising multidisciplinary research from basic sciences to medical applications and entails a close collaboration between clinicians and basic scientists across institutes. It is further defined as a two-way road: bench-to-bedside and bedside-to-bench [2], to translate discoveries from the bench into clinical application and/or the translation of clinical findings into the understanding of molecular mechanisms. CTM was emphasized to play a unique and critical role in optimizing new biotechnologies, improving clinical application of new therapeutic concepts, and ultimately improving the quality of life for patients [3]. The emergence of translational science highlights the unifying framework that bridges the continuum of knowledge creation and deployment, converting fundamental discoveries to human application, advancing the information into clinical practice, disseminating best clinical practices into communities and, ultimately, modifying the behavior of populations to improve global health [4].

It is of great significance to promote CTM among clinicians, basic researchers, biotechnologists, politicians, ethicists, sociologists, or investors and coordinate these efforts among different countries [5]. CTM as an inter-disciplinary science is developing widely and become a global priority. The 2012 Sino-American Symposium on Clinical and Translational Medicine (SAS-CTM) as one of milestone conferences on CTM was organized by Chinese Academy of Engineering, Chinese Academy of Medical Sciences, The U.S. National Institutes of Health Clinical Center, and GlobalMD Organization. The SAS-CTM was established as a bridge between China and the U.S. to exchange ideas on clinical and translational research, built up the highest level and most influential collaboration platform, and attract the special attention from leadership and opinion leaders of academies, institutions, societies, communities, governments, investors, or regulatory offices. A number of important issues on public health, clinical research, drug development, or collaborations were addressed and discussed.

## Improvement of public health

The health care and medication in China faces more challenges, with the development of economics and transform of the society, even though living conditions, food and nutrition, and the health care system have been improved during last three decades. Communicable diseases frequently seen in developing countries remain a heavy burden, while chronic diseases commonly found in developed countries are also the leading cause of death and disability in China. The development of translational medicine in China to improve the disease control and health solutions becomes even more necessary and critical. Dr. Daiming Fan, Vice President of Chinese Academy of Engineering, described China’s national 12^th^ Five-Year Plan during 2011 and 2015 entitled “*Translational Medicine in China: Improving Public Health”* that China should increase the average life expectancy by one year and reach 74.5 years old by 2015. CTM becomes the engine or driver to reform the health care system and the increase of proposed life expectancy, since the new round of the health reform in China was re-initiated in 2009.

A great number of translational research centers have been established in China since 2005 and the potential of CTM has never been such greater in China. For example, at least seven large Institutes or Centers for translational medicine were established in China during the first six months of 2010, among which the Union Center for Translational Medicine was considered a further milestone toward the development of translational medicine in China as pronounced by Dr. Zhu Chen, Minister of Ministry of Health of China [6]. Currently, CTM in China is focused predominantly on cancer, acute and chronic diseases, or common and widespread infections. As part of translational medicine initiative, China has supported a large number of CTM projects during the 11^th^ and 12^th^ Five-Year Plans through National High-Tech R&D Program of China (863 Program), 973 projects and National Nature Science Foundation. Challenges should be still faced and overcome to promote translational medicine and health care reform in China as in other countries, including how to combine translational medicine with basic research or clinical medicine and public health, better serve the health care reform and develop traditional Chinese medicine through CTM, or evaluate the impacts of centers, institutes and programs of CTM. Dr. Fan suggested that CTM-related policies, laws and regulations, resource standardization and sharing should be highly emphasized and CTM should be realized as a revolutionary opportunity for China.

## Improvement of clinical research

Clinical research has been considered as a risky business with high cost, long timelines to drug discovery, and complex regulatory components to assure a safe approach to drug and product development. Clinical research is also one of important steps in CTM and plays a critical role in translating discoveries and novel findings into new and better treatments and putting science to benefit health care reform [7]. The number of new drugs in drug discovery and development halved roughly every 9 years since 1950, falling around 80-folds in inflation-adjusted terms [8]. Toxicity is a common reason for the frustrating decline in the development of new therapeutics [9]. Dr. John I. Gallin, Director of the NIH Clinical Center, emphasized the importance of clinical research and the *New Infrastructure to Support Clinical Research*. Major initiatives have been launched at the NIH, e.g. investing in new technology to lower DNA sequencing costs, building a genetic testing registry, developing a cancer genome atlas to chart complex pathways leading to cancer, expanding research in induced pluripotent stem cells, discovering new ways to predict drug safety and opening a National Center for Translational Sciences to study the bottlenecks to discovery. The NIH Clinical Center, the largest hospital in the world totally dedicated to clinical research, is a centerpiece of the NIH clinical research infrastructure that focuses on study of pathophysiology of disease, first in human studies of new drugs and devices, and study of patients with rare diseases. Rare diseases are estimated to affect 6 - 8% of the population with an increased emphasis on study of those patients to better understanding diseases. Dr. Gallin emphasized CTM opportunities being pursued to make the road from the discovery to clinical medicine safer, faster, and less tortuous through applying high throughput technologies to understand fundamental biology, and to uncover the cause of specific diseases.

## Improvement of drug development

CTM as a new function within the pharmaceutical industry R&D organization has been applied to improve the predictability and success of drug discovery and development [10]. Disease-specific biomarker, network biomarkers and dynamic network biomarkers as the part of the CTM strategy have more value in patient selection and drug monitoring e.g. pharmacodynamics, drug efficacy and safety, target identification and validation, or compound-target interaction [11-13]. Risk factors e.g. genetics, environment, and behavior [14] attribute to differences between patients and affect clinical outcomes. Dr. Richman, Vice President, Research and Development-Translational Sciences, explained the role of CTM in drug discovery and development with a special statement on *Developing Innovative Targeted Therapies for China*. MedImmune is a 25-year-old international biotechnology company and was acquired by AstraZeneca in 2007 and became the biologics arm of AstraZeneca with a mission of helping patients with significant unmet medical need by developing innovative medicines.

The vision of personalized healthcare, treating the right patient with the right drug at the right dose, becomes more and more important. Many more programs across different therapeutic areas have applied personalized healthcare approaches as an approach of CTM. Fundamental to the goal of developing personalized healthcare is gaining deep understanding of diseases across different populations and specifically in Asia. MedImmune used SAS-CTM opportunity to present rich pipelines across five therapeutic areas including oncology, respiratory and inflammation, infectious diseases, neurosciences, cardiovascular and gastrointestinal diseases. This is one of efficient ways for pharmaceuticals to promote their innovations and developments to academic institutions, government agencies and industry partners. An example of working with scientists/physicians in China develops medications that target the Asian/Chinese patient population. MedImmune set up an exciting collaboration with Chinese hospitals to develop a richly annotated database of lung cancer and hepatocellular cancer patient samples and aid in identifying novel drug targets and developing therapeutic strategies to aid patients. Dr. Richman emphasized that strong commitment to developing targeted therapies tailored to Chinese patients requires understanding disease heterogeneity in China and building partnerships and strong scientific collaborations with Chinese key opinion leaders. Applying personalized healthcare approaches in programs across different therapeutic areas is the major strategy to improve drug discovery and development.

## Improvement of collaborations

Interdisciplinary and collaborative research is a critical part of CTM to improve the understanding of CTM researchers' needs and build useful and useable supporting and comprehensive systems. Collaborations could narrow down the available design choices for assisting CTM researchers and identify potential deficiencies of well-known theoretical frameworks used. Dr. Robert M. Califf, Vice Chancellor for Clinical and Translational Research, Duke University, addressed the *Rationale for Key Elements of Sino-American Collaboration in Clinical Research*. Health improvements seen both for individuals and populations are fully dependent upon the quality of validation of new scientific discoveries within the CTM research continuum and strong evidence of benefits and risks associated with therapeutic interventions. However, there is still a massive shortage of evidence to support decisions about health and healthcare. Less than 15% of major medical decisions are supported by high-quality evidence [15], due to the limit of geography and access to expertise during clinical investigation. International collaboration, registration of all trials and a focus on the most important human research were suggested as critical factors to close such gap.

Multinational studies are required and developed to generate evidence of CTM relevant to particular biological and cultural contexts [16], on basis of genetic and practical environments in diseases. The investigation of diseases and development of treatments does not abide by national boundaries owing to modern informatics and information technology. These new capabilities expand the concept of human biomedical research from an activity conducted in a limited number of specialized centers to a global activity accessible to all patient populations and qualified practitioners [17]. With appropriate informatics support, shared protocols, and facilitative cultural elements, common diseases can be studied on a larger scale and clinical trials in rare diseases will be able to accrue adequate sample sizes. Dr. Califf demonstrated that the number of qualified individuals in the CTM workforce and broad-scale collaboration will determine the limits on knowledge generation. In order to better capitalize on novel technological advances in promoting collaborations, five key programs specific to clinical and translational research, including clinical research training, epidemiology and global health, biostatistics, medical informatics, and health sector management, will need to train and educate a vast workforce over the coming decade.

**Figure 1:**
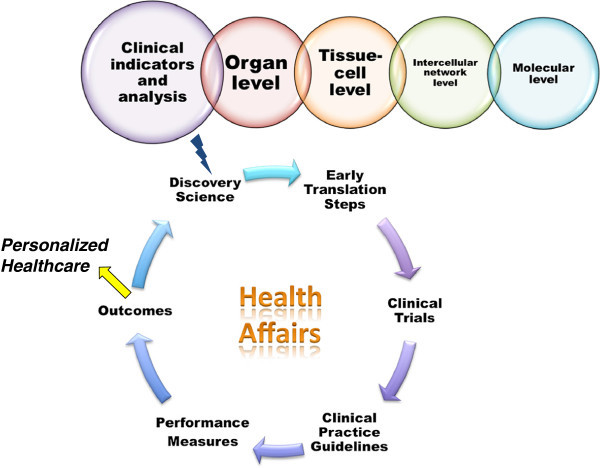
**Biological systems are complex and multidimensional and require a systems approach.** Disease and drug action originate at levels of cellular and molecular components, while physiological effects (e.g. symptoms, drug action) are at organ levels. Clinical and translational medicine should optimally link all circles and processes, e.g. discovery science, early translation steps, clinical trials, clinical practice guidelines, performance measures, and outcomes together.

In conclusion, health systems globally face challenges and opportunities in balancing quality, access, and cost, where CTM should play more important and powerful roles in the identification, development and validation of solutions and strategies. High throughput technologies applied in clinical research can reveal causes of specific diseases. Putting major emphasis on personalized healthcare approaches is one of efficient ways for pharmaceuticals to promote drug discovery and development. With a vast number of qualified individuals in the clinical and translational research workforce, strategic collaboration can gather global strengths and resources and improve health systems, care delivery, regulations and policies. CTM-driven innovation and development has the potential to achieve step-change improvements across three dimensions, as explained in Figure 1. Thus, we have the reasons to believe that CTM will play even more roles in the development of new diagnostics, therapies, healthcare, and policies and SAS-CTM will become more and more important platform to obtain the latest development in CTM internationally and explore new opportunities in the international collaborations.
